# Predicting complex traits using a diffusion kernel on genetic markers with an application to dairy cattle and wheat data

**DOI:** 10.1186/1297-9686-45-17

**Published:** 2013-06-13

**Authors:** Gota Morota, Masanori Koyama, Guilherme J M Rosa, Kent A Weigel, Daniel Gianola

**Affiliations:** 1 Department of Animal Sciences, University of Wisconsin-Madison, Madison, WI, USA; 2 Department of Mathematics, University of Wisconsin-Madison, Madison, WI, USA; 3 Department of Biostatistics and Medical Informatics, University of Wisconsin-Madison, Madison, WI, USA; 4 Department of Dairy Science, University of Wisconsin-Madison, Madison, WI, USA

## Abstract

**Background:**

Arguably, genotypes and phenotypes may be linked in functional forms that are not well addressed by the linear additive models that are standard in quantitative genetics. Therefore, developing statistical learning models for predicting phenotypic values from all available molecular information that are capable of capturing complex genetic network architectures is of great importance. Bayesian kernel ridge regression is a non-parametric prediction model proposed for this purpose. Its essence is to create a spatial distance-based relationship matrix called a kernel. Although the set of all single nucleotide polymorphism genotype configurations on which a model is built is finite, past research has mainly used a Gaussian kernel.

**Results:**

We sought to investigate the performance of a diffusion kernel, which was specifically developed to model discrete marker inputs, using Holstein cattle and wheat data. This kernel can be viewed as a discretization of the Gaussian kernel. The predictive ability of the diffusion kernel was similar to that of non-spatial distance-based additive genomic relationship kernels in the Holstein data, but outperformed the latter in the wheat data. However, the difference in performance between the diffusion and Gaussian kernels was negligible.

**Conclusions:**

It is concluded that the ability of a diffusion kernel to capture the total genetic variance is not better than that of a Gaussian kernel, at least for these data. Although the diffusion kernel as a choice of basis function may have potential for use in whole-genome prediction, our results imply that embedding genetic markers into a non-Euclidean metric space has very small impact on prediction. Our results suggest that use of the black box Gaussian kernel is justified, given its connection to the diffusion kernel and its similar predictive performance.

## Background

Prediction of yet-to-be observed phenotypes for complex quantitative traits in agricultural species [1,2] or for disease status in medicine [3] exploits connections between phenotypes, genealogies, and DNA variations potentially representing functional diversity of organisms. Systems biology approaches have uncovered abundant epistasis in model organisms, including the mouse and the rat [4], *Drosophila melanogaster*[5], and *Saccharomyces cerevisiae*[6]. In this context, Loewe [7] proposed an evolutionary systems biology framework for arriving at a better understanding of molecular interactions, given that epistatic interactions between mutations are commonly observed. Therefore, it seems reasonable to argue that genotypes and phenotypes may be connected in forms that are not well addressed by the linear additive models that are standard in quantitative genetics. Bayesian regularized parametric linear additive smoothers, e.g., [8,9] may not be fully adequate for capturing genetic signals under epistatic scenarios [10,11]. Furthermore, attempts to account for epistasis by including interactions in a linear model produces a highly parameterized model structure, possibly yielding a poor predictive ability in cross-validation, and which does not scale well if high-order interactions are included in the model.

Genetic risk prediction in medicine relies on using genomic information to predict the chance of contracting a disease, for example, in personalized medicine for preventive treatment and clinical health care. Prediction of genetic risk derived from pre-selected marker variants is mainstream in this domain, as opposed to prediction based on fitting whole-genome markers simultaneously, as done with great success in animal and plant breeding [8-11]. However, the variants detected in this way are typically not useful for genetic risk prediction, because they explain only a small fraction of the total genetic variance as estimated from covariances between relatives, for example using twin and family studies. Moreover, it has been shown that a large number of variants that do not reach genome-wide statistical significance contribute to the total additive genetic variance [12].

Development of statistical models to predict phenotypic outcomes from all available molecular information that are capable of capturing complex genetic network architectures is therefore important. Arguably, a good predictive model should account for most of the genetic variability, as well as reflect the underlying genetic architecture properly. Also, a predictive model should be flexible with respect to type of input data, e.g., high-throughput chip-based genotypes or whole-genome sequences, and mode of gene action.

An appealing alternative is provided by a kernel-based parametric method known as BLUP (Best Linear Unbiased Prediction) of genetic effects, developed in the 1950’s by C. R. Henderson, an animal breeder [13]. BLUP can also be viewed as a regression of a phenotype on a pedigree-based relationship matrix **A** (when the model is additive), and it has been used for genetic improvement of livestock species for decades. This method was recently extended to incorporate SNP (single nucleotide polymorphisms) by replacing **A** by a genomic relationship matrix **G**[14], although there is no impediment to using **A** and **G** together [15]. BLUP is suited for handling a massive amount of genetic information, because the computational burden can be proportional to the number of data points rather than the number of predictor variables (e.g., markers), and this is particularly true if a common weight is assigned to a each marker. Recently, kernel-based non-parametric models e.g., [15-18] have been proposed. A non-parametric treatment can accommodate nonlinear dependencies of phenotypes on predictor variables without explicitly modeling them. This suggests that these procedures can potentially pick up various forms of gene action without posing richly parametrized structures that require making strong distribution and genetic architecture assumptions a priori [10,15]. For example, Long et al. [16] used a computer simulation and found that the predictive ability of a non-parametric smoother was superior to that of a parametric linear counterpart when non-additive effects were strong. These authors also gave evidence that non-parametric smoothing is competitive to linear smoothing, even when additivity accounts for most of the total genetic variability.

Kernel ridge regression [19], a kernel generalization of standard ridge regression [20], is also a non-parametric smoothing method. Ridge regression has received some attention in quantitative genetics in the context of mixed linear models [10,15,21-24], and the non-parametric version is carried out by constructing a spatial distance-based relationship matrix called kernel, as opposed to using additive genomic relationship kernels, **A** or **G**, which only embed correlations due to additive genetic effects. The choice of a kernel is equivalent to modeling covariance structure among individuals, and phenotypes are regressed on this kernel to obtain estimates of non-parametric regression coefficients.

A simulation study [18] found that in the presence of non-additive effects, a spatial distance-based kernel can outperform an additive genomic relationship kernel in predictive performance, but this has not been explored thoroughly with real data. Furthermore, while the set of all SNP genotype configurations on which a model is built is finite, past research has employed spatial distance-based kernels with infinite, unbounded domains, such as the Gaussian kernel. Our first objective in this study is to compare a spatial distance kernel with a non-spatial distance kernel. Secondly, we assess the performance of a non-Gaussian spatial distance kernel by deploying kernels on graphs as the choice of a basis function, a procedure that is suitable for discrete input data structures. Instead of encoding SNP data in a continuous Euclidean space, as in the case of the Gaussian kernel, we investigated kernels on a non-Euclidean space. We examined a diffusion kernel proposed by Kondor and Lafferty [25], Smola and Kondor [26] and Lafferty and Lebanon [27], which is a kernel defined for functions on discrete spaces, such as a graph. A brief review on ’kernels on graphs’ is given by [28], and “graph kernels” are discussed in [29]. As shown later, the diffusion kernel can be viewed as a discretization of the Gaussian kernel. We also tested the sensitivity of applying the same bandwidth parameter to autosomes and allosomes in the spatial distance kernels.

This paper investigates the use of several kinds of kernels in a kernel ridge regression framework for genome-assisted prediction of quantitative traits. Two data sets representing dairy cattle and wheat were employed for this purpose. The paper is organized as follows. In the Methods section, we describe the data and introduce basic notions of kernel ridge regression. We then apply the diffusion kernel to strings of dummy variable marker sequences; the motivation of the non-Euclidean metric space is followed by an introduction of the diffusion kernel. In the next section, main results are presented. Finally, in the Discussion section, we address the implication of our results and make concluding remarks.

## Methods

### Data

Dairy cattle and wheat data were used. The dairy data was provided by the USDA-ARS Animal Improvement Programs Laboratory (Beltsville, MD) and represented 7902 Holstein bulls, each with 43 134 SNPs (minor allele frequency > 0.025) spanning across the whole genome. The target response variable was progeny test predicted transmitting ability (PTA) of productive life (PL). PL is a measure of the observed length of time that a cow stays in the herd, from first calving to culling, and PTA is an estimate of half of the breeding value of a bull, which is a smoothed average assuming additive inheritance. PL is lowly heritable, with heritability estimated at 0.1 [30]. The genotype for each of 42 438 SNP loci on autosomes was coded as 0 (homozygous for allele “a”), 1 (heterozygous), or 2 (homozygous for allele “A”), according to the number of copies of the “A” allele. The remaining 696 loci on the X chromosome were coded as either 0 or 2, representing absence or presence of the “A” allele, respectively. Missing genotypes, due to either low call rates for some SNPs or poor DNA quality, were imputed via random sampling of genotypes with probabilities corresponding to observed genotype frequencies at each locus. Note that other more precise methods are available but were not used here.

The wheat data included 599 inbred lines collected by the International Maize and Wheat improvement Center in Mexico (CIMMYT). Each line was genotyped with 1279 Diversity Array Technology (DArt) markers generated by Triticarte Pty. Ltd. These binary markers take the form of presence (1) or absence (-1). The phenotype here was average grain yield of each line in the first out of four environments represented in the data set, scaled to have zero mean and variance one. Missing genotypes were imputed as for the cattle data above. This data set has been also analyzed with support vector regression and neural network methods [17,31].

### Kernel ridge regression

Our goal is to predict an unobserved response y, for example PL in R from a vector of genotypes **x** at a large number of SNP loci; when *p* SNP are considered, **x** is in $Z_{3}^{p}$. To this end, we would like to establish a function $g:Z_{3}^{p}\to R$ mapping sequences of SNP genotypes onto the real line. A general setting is:

$$y_{i}=g(x_{i})+\epsilon_{i},$$

where *y*_*i*_ is a response variable on case *i*(*i*=1,2,…,*n*), **x**_*i*_ is a *p*×1 vector of genotypes obtained on *i*, *g*(**x**_*i*_) is a genetic effect interpretable as the conditional expectation function *g*(**x**_*i*_)=*E*(**y**_*i*_|**x**=**x**_*i*_), and *ϵ*_*i*_ is a residual.

We use kernel ridge regression to infer the unknown function *g*, and select an appropriate kernel K via a reproducing kernel Hilbert space ℋ (RKHS) of functions on $Z_{3}^{p}$, and optimize:

(1) $$∥y-g{∥}^{2}+\lambda ∥g{∥}_{ℋ}^{2}$$

with respect to **G**, where the first term is the residual sum of squares, $∥g{∥}_{ℋ}^{2}$ is the squared norm of *g* under a Hilbert space, and *λ* is a regularization parameter. The representer theorem [32] is used to find the optimal *g*.

In non-parametric regression, the search space is infinite, but the representer theorem allows confining the search to a specific set of functions. It has been shown [10,15,24,32] that the optimizer will be in the span of the functions indexed by the observed covariates, and that the problem simplifies to optimization of:

$$\ell (\alpha |\lambda )=∥y-K\alpha {∥}^{2}+\lambda ∥K\alpha {∥}_{ℋ}^{2},$$

where **K**={*K*(*i*,*j*)=*K*(*x*_*i*_,*x*_*j*_)} is a *n*×*n* symmetric positive (semi) definite matrix; ***α*** is an unknown *n*×1 vector of non-parametric regression coefficients; and **g**=**K*α***, is the function that minimizes (1). By properties of a reproducing kernel, $∥K\alpha {∥}_{ℋ}^{2}=\alpha^{\prime }K\alpha$, so that the function to be minimized with respect to ***α*** is:

(2) $$\begin{array}{ll} \ell (\alpha |\lambda )={\left(y-K\alpha \right)}^{\prime }(y-K\alpha )+\lambda \alpha^{\prime }K\alpha . & \; \end{array}$$

This is equivalent to writing:

y=Kα+ϵ

and then maximizing a penalized likelihood. This penalized likelihood is obtained by assuming that *p*(*y*|***α***,*σe*2) and that ***α*** follows $N(0,K^{-1}\sigma_{\alpha }^{2})$, where $\sigma_{e}^{2}$ is the variance of the residuals, and $\sigma_{\alpha }^{2}$ is a variance component.

Next, we review additive genomic relationship kernels and the Gaussian kernel, and then present how one can build a kernel on a graph with discrete inputs. Hereafter, we denote **K** as the kernel matrix indexed by the observed covariate; and *K*(*i*,*j*) indicates particular elements of **K**; K is the infinite dimensional Gaussian kernel, or the 3^*p*^×3^*p*^ dimensional kernel for the diffusion kernel.

### Additive genomic relationship kernels

Two types of additive genomic relationship kernels were tested in this study. First, an additive genomic relationship matrix (**G1**) was constructed following VanRaden [14] as:

$$\begin{array}{lll} G1 & =\frac{{\text{ZZ}}^{\prime }}{2\sum p_{j}(1-p_{j})},\; & \; \end{array}$$

where **Z**={*Z*_*ij*_} is a *n*×*p* matrix of centered SNP marker codes, with the entry for *i*th individual and the *j*th marker taking the form

$$\begin{array}{l} Z_{\text{ij}}=\left\{\begin{array}{ll} 0-2p_{j} & \text{if homozygous for “a”}\\ 1-2p_{j} & \text{if heterozygous}\\ 2-2p_{j} & \text{if homozygous for “A”}. \end{array}\right) \end{array}$$

Here *p*_*j*_ is the frequency of allele “A” computed from a base population. The denominator of **G1** is a scaling parameter. In practice, the allele frequencies are estimated from the data at hand, but this yields semi-positive definite matrices as discussed by Strandén and Christensen [33].

A second additive genomic relationship matrix (**G2**) was also as in VanRaden [14]

$$\begin{array}{ll} G2=\frac{{\text{WW}}^{\prime }}{p}, & \; \end{array}$$

where **W** is a matrix of standardized genotypes [34] with its *j*th column being

$$\begin{array}{lll} w_{\mathrm{.j}} & =\frac{z_{\mathrm{.j}}}{\sqrt{2p_{j}(1-p_{j})}},\; & \; \end{array}$$

where **z**_.*j*_ is the *j*th column of **Z** and *p* represents the number of SNPs.

Since the Holstein data set led to non-positive **G1** and **G2** matrices because of numerical issues, **G**_*i*_(*i*=1,2) was modified to $G_{i}^{*}=0.95G_{i}+0.05I$, yielding **G**^∗^ matrices that provided valid kernels. This may also avoid numerical instability in the eigenvalue decomposition of the kernel as explained later. The wheat data produced semi-positive definite genomic relationship kernel matrices. The kernel **G1** has been applied to several inbred line populations in the past, e.g., Ober et al. [35].

### Gaussian kernel

In a Gaussian kernel, the distance between a pair (*i*,*j*) of genotypes is represented as a squared Euclidean norm. Given a positive bandwidth parameter *θ*, the kernel takes the form

$$\begin{array}{lll} K(x_{i},x_{j}) & =\exp(-\theta \overset{2}{\underset{\text{ij}}{d}})\; & \;\\ & =\overset{p}{\underset{k=1}{\prod }}\exp(-\theta {\left(x_{\text{ik}}-x_{\text{jk}}\right)}^{2}),\; & \; \end{array}$$

where $d_{\text{ij}}=\;\sqrt{{\left(x_{i1}\;-x_{j1}\right)}^{2}\;+\;\cdots \;+\;{\left(x_{\text{ik}}-x_{\text{jk}}\right)}^{2}\;+\;\cdots \;+\;{\left(x_{\text{ip}}-x_{\text{jp}}\right)}^{2}}$, and *x*_*ik*_ (*i*,*j*=1,…,*n*,*k*=1,…,*p*) is the SNP genotype for individual *i* at SNP *k*. A small Euclidean distance between two individuals reflects a strong similarity in state between their genotypes. On the one hand, as *θ* increases, the kernel evaluation approaches *K*(**x**_*i*_,**x**_*j*_)=0, producing a “sharp” or “local” kernel. On the other hand, as *θ*→0, the kernel approaches 1, that is, a situation where the two individuals “match” perfectly, providing a “global” kernel.

### Non-Euclidean metric space

The SNP data on *p* loci on some individual often come as $x=(x_{1},x_{2},\ldots ,x_{p})\in Z_{3}^{p}$, which is clearly a discrete space, as there are 3^*p*^ possible configurations of genotypes (not all of which are observable). Before defining the diffusion kernel, consider the meaning of ’diffusion on a graph’. Suppose *p*=1, and consider a function *k*_*x*_ that measures the spread of ’influence’ of the genotype at this locus over the other possible genotypes by assuming that the ’influence’ diffuses like heat dissipates. Let $k_{\tilde{x}}(0,x)=1_{x=\tilde{x}}(x)$, be the indicator function for genotype $\tilde{x}$ on $Z_{3}$. We call this the time 0 diffusion, since in this case $\tilde{x}$ has absolutely no influence on other genotypes; that is, the influence of $\tilde{x}$ does not diffuse out to its neighbors. Now, define the time *t* diffusion of the ’influence’ of genotype $\tilde{x}$ on genotype *x* to be:

(3) $$\begin{array}{ll} \;k_{\tilde{x}}(t,x)=k_{\tilde{x}}(t-1,x)+\underset{|x-x^{\prime }|=1}{\sum }\alpha [k_{\tilde{x}}(t-1,x^{\prime })-k_{\tilde{x}}(t-1,x)], & \; \end{array}$$

where *α* is a constant rate of diffusion and each summand is the differential gradient of the ’influence’ between genotypes *x* and *x*^′^. This is illustrated in Table 1. As stated above, there is no diffusion at *t*=0. Subsequently, the time 1 diffusion with *α*=0.1 when $\tilde{x}=1$ is computed as:

$$\begin{array}{l} k_{1}(1,x=0)=k_{1}(0,x=0)+\alpha [k_{1}(0,x^{{}^{\prime }}=1)-k_{1}(0,x=0)]\\ \;=0+0.1[1-0]\\ \;=0.1\\ k_{1}(1,x=1)=k_{1}(0,x=1)+\alpha [k_{1}(0,x^{{}^{\prime }}=0)-k_{1}(0,x=1)]\\ \;+\alpha [k_{1}(0,x^{{}^{\prime }}=2)-k_{1}(0,x=1)]\\ \;=1+0.1[0-1]+\;0.1[0-1]\\ \;=0.8\;\\ k_{1}(1,x=2)=k_{1}(0,x=2)+\alpha [k_{1}(0,x^{x^{\prime }}=1)-k_{1}(0,x=2)]\\ \;=0+0.1[1-0]\\ \;=0.1 \end{array}$$

**Table 1:** Example of diffusion on a graph

***α*= 0.1**				***α* = 0.2**				***α* = 0.2**			
***x* =**	**0**	**1**	**2**	***x*=**	**0**	**1**	**2**	***x*=**	**0**	**1**	**2**
*k*_1_(0,*x*)	0	1	0	*k*_1_(0,*x*)	0	1	0	*k*_2_(0,*x*)	0	0	1
*k*_1_(1,*x*)	0.1	0.8	0.1	*k*_1_(1,*x*)	0.2	0.6	0.2	*k*_2_(1,*x*)	0	0.2	0.8
*k*_1_(2,*x*)	0.17	0.66	0.17	*k*_1_(2,*x*)	0.28	0.44	0.28	*k*_2_(2,*x*)	0.04	0.28	0.68
*k*_1_(3,*x*)	0.219	0.562	0.219	*k*_1_(3,*x*)	0.312	0.376	0.312	*k*_2_(3,*x*)	0.171	0.330	0.498
*k*_1_(15,*x*)	0.331	0.336	0.331	*k*_1_(15,*x*)	0.333	0.333	0.333	*k*_2_(15,*x*)	0.324	0.333	0.342

***x*** = (0, 1, 2) are genotype codes; ***α*** = (0.1, 0.2) is the diffusion rate; $k_{\tilde{x}}(t,x)$ is the time ***t*** diffusion of the influence of genotype $\tilde{x}$ on genotype ***x***.

As shown in Table 1, as *t* increases the ’influence’ spreads over all genotypes more evenly; also, the larger *α* is, the faster the diffusion is with respect to time *t*.

Writing (3) in vector form, with $k_{\tilde{x}}(t,x)=k_{\tilde{x}}(t)$, we get:

(4) $$\begin{array}{lll} {\;k}_{\tilde{x}}(t) & =k_{\tilde{x}}(t-1)+\alpha Hk_{\tilde{x}}(t-1)\; & \;\\ & =(I+\alpha H)k_{\tilde{x}}(t-1)\; & \;\\ & ={\left(I+\alpha H\right)}^{t}k_{\tilde{x}}(0),\; \end{array}$$

where **I** is a 3×3 identity matrix; $k_{\tilde{x}}(0)$ is a constant 3×1 matrix of initial values, and

(5) $$\begin{array}{ll} H=\left[\begin{array}{lll} -1 & 1 & 0\\ 1 & -2 & 1\\ 0 & 1 & -1 \end{array}\right] & \; \end{array}$$

with the first, second, and third rows of the **H** matrix corresponding to *k*_0_,*k*_1_, and *k*_2_ respectively. The negative of this matrix is called the Laplacian of a graph Γ, given by:

(6) $$\begin{array}{ll} 0-1-2. & \; \end{array}$$

Let Γ be an undirected graph with vertex set *V*(Γ). In general, the Laplacian of a graph Γ, *L*(Γ), is a *V*(Γ) dimensional square matrix given by

$$\begin{array}{lll} \;L(\Gamma ) & =-H(\Gamma )\; & \;\\ & =-A(\Gamma )+\Lambda ,\; & \; \end{array}$$

where **A** is an adjacency matrix and ***Λ*** is a diagonal matrix with $\Lambda_{\text{ii}}=\overset{n}{\underset{j=1}{\sum }}A_{\text{ij}}$. We can therefore generalize this ’diffusion’ for any graph Γ by using **H**(Γ)=−**L**(Γ). Under this definition, given any *V*(Γ) dimensional vector **W**,

$$w^{t}H(\Gamma )w=-\underset{i∼j}{\sum }{\left(w_{i}-w_{j}\right)}^{2}\leq 0,$$

which shows that **H**(Γ) is a negative semi-definite matrix.

The most naive way of constructing a graph on $Z_{3}^{p}$ is a Hamming graph. For the case *p*=1, a Hamming graph is simply a complete graph of size 3, and has the form

(7) $$\begin{array}{ll} \begin{array}{lll} 0 & - & 1\\ \;\setminus & \;/\\ \;2 \end{array} & \; \end{array}$$

On this graph, the distance from genotype 0 (’*aa*’) to genotype 2 (’*AA*’) is the same as that from 0 (’*aa*’) to 1 (’ *Aa*’). Since genotype ’*aa*’ has no copies of the ’*A*’ allele, it may be more reasonable to assume that genotype ’ *Aa*’ is closer to ’*AA*’, which has two copies of the ’A’ allele. This can be viewed from a mutational perspective as well. Genotype 0 (’*aa*’) requires two mutations to become genotype 2 (’*AA*’), while genotype 1 (’ *Aa*’) requires only one mutation. Thus, the graph of interest would be given by (6). The latter is a path graph for SNP data, which will be taken as a minimal basis for our graph. In a path graph, all vertices are on a straight line, as in (6).

A SNP grid of *p* loci is a *p* dimensional grid with vertices in $Z_{3}^{p}$, with two vertices **x** and **x**^′^ being adjacent if and only if

$$\overset{p}{\underset{i=1}{\sum }}|x_{i}-x_{i}^{\prime }|=1.$$

For example, the graph below is the grid for 2 loci derived from the Cartesian graph product of two path graphs as in (6):

(8) $$\begin{array}{ll} \begin{array}{lllll} 02 & - & 12 & - & 22\\ \;| & \;| & \;|\\ 01 & - & 11 & - & 21\\ \;| & \;| & \;|\\ 00 & - & 10 & - & 20 \end{array} & \; \end{array}$$

**Figure 1:**
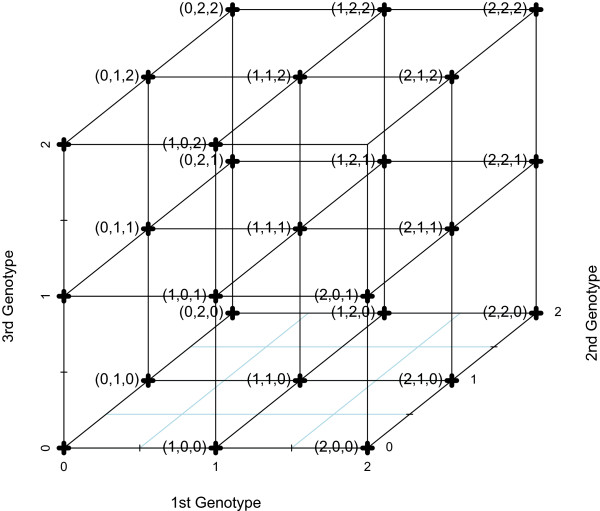
**A SNP grid graph.** A SNP grid graph with 3 genotypes (*p* = 3). It has 3^3^=27 vertices.

The graph Laplacian for graph (8) is a square matrix of dimension 3^2^×3^2^:

$$\begin{array}{lll} \;L(\Gamma ) & =-H(\Gamma )\; & \;\\ & =\left[\begin{array}{lllllllll} 2_{00} & -1 & 0 & -1 & 0 & 0 & 0 & 0 & 0\\ -1 & 3_{01} & -1 & 0 & -1 & 0 & 0 & 0 & 0\\ 0 & -1 & 2_{02} & 0 & 0 & -1 & 0 & 0 & 0\\ -1 & 0 & 0 & 3_{10} & -1 & 0 & -1 & 0 & 0\\ 0 & -1 & 0 & -1 & 4_{11} & -1 & 0 & -1 & 0\\ 0 & 0 & -1 & 0 & -1 & 3_{12} & 0 & 0 & -1\\ 0 & 0 & 0 & -1 & 0 & 0 & 2_{20} & -1 & 0\\ 0 & 0 & 0 & 0 & -1 & 0 & -1 & 3_{21} & -1\\ 0 & 0 & 0 & 0 & 0 & -1 & 0 & -1 & 2_{22} \end{array}\right]\; & \; \end{array}$$

where the subscripts denote the vertices of graph (8). When there are *p* loci, the *p*-dimensional grid graph has 3^*p*^ vertices corresponding to sequences of genotypes, such that two vertices are adjacent if and only if just one SNP locus differs by 1. Now, suppose *p*=3. The Cartesian graph product of (6) and (8) yields a 3 dimensional grid graph with 3^3^ vertices, as shown in Figure 1. The diffusion kernel computes a similarity between two vertices on this graph, and projects this information into a more interpretable space.

### Diffusion kernel on a non-Euclidean metric space

Consider now the continuous analog of the diffusion scheme above. This can be done by making ‘time’ or ‘space’ continuous, and ‘time’ will be made continuous first. Let *α*=*θh* (*θ*>0) and *t*=1/*h*. By using a small *h*, we can achieve a discretization of the ‘diffusion time’ on a much finer scale, and the coefficient matrix is:

(9) $$\begin{array}{ll} {\left(I+\mathrm{\theta h}H(\Gamma )\right)}^{1/h}. & \; \end{array}$$

If an infinitesimal scale is considered by taking *h*→0, (9) converges to:

(10) $$\begin{array}{lll} \underset{h\to 0}{\;\lim}{\left(I+\mathrm{\theta h}H(\Gamma )\right)}^{1/h} & =\exp(\theta H)\; & \;\\ & =\overset{\infty }{\underset{k=0}{\sum }}\frac{\theta^{k}}{k!}H^{k}=I+\theta H+\frac{\theta^{2}}{2}H^{2}\; & \;\\ & +\frac{\theta^{3}}{3!}H^{3}+\cdots +\frac{\theta^{n}}{n!}H^{n}+\cdots \; \end{array}$$

If a graph Γ with a Laplacian **L**(Γ) is considered, then exp(−*θ***L**(Γ)) is called the diffusion kernel or heat kernel for graph Γ, where *θ* is a rate of diffusion [25]. Here, putting K=exp(θH) and taking the derivative with respect to *θ* gives:

(11) $$\begin{array}{ll} \frac{d}{\mathrm{d\theta}}K=HK, & \; \end{array}$$

which is a discrete diffusion equation (heat equation) on a graph with **H**=−**L**(Γ). Note that diffusion kernels always need to be associated with a graph.

A Gaussian kernel is obtained by making this diffusion kernel “space” continuous. The connection between the two kernels is provided in Appendix A.

### Diffusion kernel indexed by observed covariates

When a graph Γ is large and asymmetric, the computation of the diffusion kernel K(Γ) can be extremely difficult. For instance, for a SNP grid with 43 134 loci, the dimension of K is 3^43134^ by 3^43134^. However, symmetry helps. If a closed form of K can be arrived at, there is no need to compute $K(x,x^{\prime })$ for all pairs of genotype sequences **x**,**x**^′^. This is indeed the case for the Gaussian kernel, where the dimension of K is infinite. With Kondor and Lafferty’s result given in [25], we may obtain the closed form of the diffusion kernel from the sample for our SNP grid.

First, one needs to consider the Cartesian graph product for the diffusion kernel of a graph. Let $K_{1}(\theta )$ and $K_{2}(\theta )$ be the kernels for graphs Γ_1_ and Γ_2_, respectively. The diffusion kernel for Γ=Γ_1_□Γ_2_ is given by [25]:

(12) $$\begin{array}{ll} K_{1}(\theta )⊗K_{2}(\theta ), & \; \end{array}$$

where □ denotes the Cartesian graph product and ⊗ is the tensor product (infinite dimensional Kronecker product). Consider a graph with one locus, Γ_0_, with form 0−1−2. Then, we see that the diffusion kernel of the SNP grid on *p* loci with bandwidth parameter *θ* is given by:

$$K_{\theta }^{⊗p}=\overset{p}{\underset{i=1}{⊗}}K_{\theta }(\Gamma_{0}).$$

To this end, we just need to compute $K_{\theta }(\Gamma_{0})=\exp(\theta H)$ with **H** in (5).

With this result, one can create the **H** matrix for a SNP grid as follows. Let **x** and **x**^′^ be SNP data for *p* loci; *n*_*s*_ be the number of loci for which |**x**_*i*_−**x***i*′|=*s*, and *m*_11_ be the number of loci for which **x**_*i*_=**x***i*′=1. In other words, *n*_1_ is the number of loci at which two individuals differ by 1, and *m*_11_ is the number of loci at which two individuals share heterozygous states. Then:

(13) $$\begin{array}{lll} {\;K}_{\theta }^{\text{snpgrid}}(x,x^{{}^{\prime }})\propto & {\left(\frac{-2e^{-3\theta }+2}{e^{-3\theta }+3e^{-\theta }+2}\right)}^{n_{1}}\; & \;\\ \;\times & {\left(\frac{e^{-3\theta }-3e^{-\theta }+2}{e^{-3\theta }+3e^{-\theta }+2}\right)}^{n_{2}}\; & \;\\ \;\times & {\left(\frac{4e^{-3\theta }+2}{e^{-3\theta }+3e^{-\theta }+2}\right)}^{m_{11}},\; \end{array}$$

with proportionality constant (*e*^−3*θ*^+3*e*^−*θ*^+2)^*q*^, where *q*=*n*_1_+*n*_2_+*m*_11_. The last term is a contribution from heterozygosity. We refer to this as SNP grid kernel, specifically developed to model SNP data in this study. A proof of this result is given in Appendix B.

### Diffusion kernel for binary genotypes

Another diffusion kernel tailored for binary genotypes is required for the chromosome X of sires or for the wheat inbred lines. In this setting, instead of (6), the path graph for one locus (*p*=1) is:

0−2

and the corresponding graph Laplacian is given by:

(14) $$\begin{array}{lll} \;L(\Gamma ) & =-H(\Gamma )\;\\ & =\left[\begin{array}{ll} 1 & -1\\ -1 & 1 \end{array}\right],\; & \; \end{array}$$

as opposed to (5). For two loci (*p*=2), the Cartesian product of graphs Γ_1_(0−2) and Γ_2_(0−2) yields the graph:

(15) $$\begin{array}{ll} \begin{array}{lll} 00 & - & 01\\ \;| & \;|\\ 10 & - & 11 \end{array} & \; \end{array}$$

where the first digits ∈*V*(Γ_1_) and the second digits ∈*V*(Γ_2_). Then, the associated graph Laplacian is:

$$\begin{array}{lll} L(\Gamma ) & =-H(\Gamma )\; & \;\\ & =\left[\begin{array}{llll} 2_{00} & -1 & -1 & 0\\ -1 & 2_{01} & -1 & 0\\ -1 & 0 & 2_{10} & -1\\ 0 & -1 & -1 & 2_{11} \end{array}\right],\; & \; \end{array}$$

where the subscripts denote the rows and columns of vertices of graph (15). Specifically, we compute **K**_*θ*_= exp(*θ***H**) with **H** defined in (14) and perform the tensor product *p* times. With this, the kernel is given by:

(16) $$\begin{array}{ll} K_{\theta }^{\text{hypercube}}(x,x^{\prime })\propto {\left(\frac{1-\exp(-2\theta )}{1+\exp(-2\theta )}\right)}^{d(x,x^{\prime })}, & \; \end{array}$$

where *d*(**x**,**x**^′^) is the Hamming distance, that is, number of coordinates at which **x** and **x**^′^ differ [25]. Following Kondor and Lafferty [25], this diffusion kernel for binary markers will be referred to as the hypercube kernel.

### Combining SNP grid kernels and hypercube kernels

In addition in the Holstein data, we combined the two kernels derived from autosomes and from chromosome X to see the influence of applying the same value of the bandwidth parameter to different types of chromosomes. This is given by:

(17) $$\begin{array}{ll} K^{\text{all}}=K^{\text{snpgrid}}\#K^{\text{hypercube}}, & \; \end{array}$$

where *#* is a Hadamard product of matrices. In general, given a set of *n* individuals, we may partition SNP into several subsets, say **x**=(**x**_1_,**x**_2_,....,**x**_*r*_). If **K**^*q*^ is the diffusion kernel corresponding to subset **x**_*q*_, then the diffusion kernel for all sets can be computed as:

$$K^{\text{all}}=K^{1}\#K^{2}\#\cdots \#K^{r}.$$

This result also holds for the Gaussian kernel, but not necessarily for every kernel, e.g., the exponential kernel defined with the Euclidean distance (||**x**_*i*_−**x**_*j*_||) does not hold this property.

### Bayesian treatment of kernel ridge regression

Once the choice of the kernel is determined, (2) can be maximized by taking the derivative of *ℓ*(***α***) with respect to ***α*** to obtain:

$$\begin{array}{ll} \hat{\alpha }={\left(K+\lambda I\right)}^{-1}y, & \; \end{array}$$

where *λ* is a regularization parameter. Here, implementation of kernel ridge regression was cast in a Bayesian framework with $\lambda =\frac{\sigma_{\epsilon }^{2}}{\sigma_{\alpha }^{2}}$, where $\sigma_{\epsilon }^{2}$ and $\sigma_{\alpha }^{2}$ are the residual variance and the variance attached to ***α*** respectively. Then, note that [36,37]:

$$\begin{array}{lll} \;\exp(-\frac{1}{2}\ell (\alpha ))= & \exp\left\{-\frac{1}{2}[{\left(y-K\alpha \right)}^{\prime }(y-K\alpha )+\lambda \alpha^{\prime }K\alpha ]\right\}\; & \;\\ \;\propto & \exp\left(-\frac{1}{2\overset{2}{\underset{\epsilon }{\sigma }}}{\left(y-K\alpha \right)}^{\prime }(y-K\alpha )\right)\; & \;\\ \;\times & \exp\left(-\frac{1}{2\overset{2}{\underset{\alpha }{\sigma }}}\alpha^{\prime }K\alpha \right).\; & \; \end{array}$$

This is proportional to $p(\alpha |y,\sigma_{e}^{2},\sigma_{\alpha }^{2})\propto p(y|\alpha ,\sigma_{e}^{2})p(\alpha |\sigma_{\alpha }^{2})$, that is, the posterior density of ***α*** (given $\sigma_{e}^{2}$ and $\sigma_{\alpha }^{2}$) for the linear model:

$$\begin{array}{ll} y=K\alpha +\epsilon , & \; \end{array}$$

with ***ϵ***∼*N*(0,**I***σe*2) and with prior ***α***∼*N*(0,**K**^−1^*σα*2). Minimizing *ℓ*(***α***) will maximize $\exp(-\frac{1}{2}\ell (\alpha ))$, so $\hat{\alpha }$ is the conditional posterior mode of ***α***. One may change the basis **K** using the eigenvalue decomposition **K**=**ΛΨΛ**^′^, where ***Λ*** is the matrix of eigenvectors of **K** and **Ψ** is a diagonal matrix in which diagonals are the eigenvalues, as shown in de los Campos et al. [36], such that, for ***δ***=**ΨΛ**^′^ one gets, in a fully Bayesian model,

$$\left\{\begin{array}{l} y=\Lambda \delta +\epsilon ,\\ p(\epsilon ,\delta ,\sigma_{\epsilon }^{2},\sigma_{\alpha }^{2})\propto N(\epsilon |0,I\sigma_{\epsilon }^{2})N(\delta |0,\Psi \sigma_{\alpha }^{2})p(\sigma_{\epsilon }^{2},\sigma_{\alpha }^{2}) \end{array}\right)$$

Once a prior is assigned to $\sigma_{e}^{2}$ and $\sigma_{\alpha }^{2}$, a Monte Carlo Markov Chain (MCMC) scheme can be used to infer all unknown parameters, including *λ*. Scaled inverse chi-square prior distributions were assigned to $\sigma_{e}^{2}$ and $\sigma_{\alpha }^{2}$, each with 3 degrees of freedom and a scale parameter equal to 1. Samples from posterior distributions were obtained by the Gibbs sampler in [36], and each of the analyses was based on 100 000 MCMC samples with the first 60 000 samples discarded as burn-in. After burn-in, samples were thinned at a rate of 10, resulting in 4000 mildly correlated samples for posterior inference. Convergence was monitored by inspecting trace plots of the variance parameters. A bandwidth parameter *θ* yielding high predictive ability is needed as well. However, sampling of the bandwidth parameter in MCMC sampling requires computation of kernels at each iteration, which is very demanding given the number of individuals and SNP considered in our study. For this reason, evaluation of the diffusion kernel was performed over a fixed grid of values of *θ*. The range of *θ* considered provided average values of $K(x,x^{{}^{\prime }})$ that were evenly spaced, approximately, between 0.13 to 0.99. Computation of kernels and Gibbs sampling was carried out in Fortran and in R, respectively.

### Assessment of predictive ability

The predictive ability of RKHS models with either a diffusion kernel or a Gaussian kernel was assessed by cross-validation. A subset of 5403 bulls born from 1952 through 2003 was used as the training set for the Holstein data. A testing set of 2499 bulls born from 2004 through 2006 was used to evaluate predictive ability. For the wheat data, a 10 fold cross-validation scheme was applied by assigning 599 lines randomly to one of 10 disjoint subsets. Each set was used for validation in turn, while the other 9 subsets were used to train the model. To illustrate, we estimated ***α*** in the Holstein data using the training set **y**=(*y*_1_,⋯,*y*_5403_)^′^ and their corresponding SNP genotypes **x**_1_,⋯,**x**_5403_, and then predicted responses in the testing set as:

$$\begin{array}{lll} {\hat{y}}^{\text{test}} & =1{\hat{\mu }}^{\text{train}}+K^{\text{test}↔\text{train}}{\hat{\alpha }}^{\text{train}},\; & \; \end{array}$$

where ${\hat{y}}^{\text{test}}$ is the 2499 × 1 vector of predicted responses of bulls in the testing set; **1** is a 2499×1 vector of ones; ${\hat{\mu }}^{\text{train}}$ is the posterior mean of the intercept estimated from the training set; **K**^*test*⇔*train*^ is a 2499 × 5403 matrix with elements *k*(*j*,*i*)^*test*⇔*train*^ representing the allelic similarity between bulls in the testing (*j*=1,…,2499) and training (*i*=1,…,5403) sets, with the same bandwidth parameter employed in the training set, and ${\hat{\alpha }}^{\text{train}}$ is the vector of posterior means of 5403 non-parametric regression coefficients obtained from the training set. In the equation above, **K**^*test*⇔*train*^ was either the diffusion or the Gaussian kernel.

In a Bayesian setting, however, one can embed all the above steps in a convenient way. Prior to Gibbs sampling, first we construct a full kernel matrix containing both training and testing data sets. We treat the responses of testing set individuals as unobserved, and these values are predicted via a predictive distribution. This is easy to incorporate in the Gibbs sampling scheme. Pearson’s correlation between the predicted values (mean of the predictive distribution) and the observed PTA, Cor( ${\hat{y}}^{\text{test}},y^{\text{PTA}})$, and predictive mean-squared error (MSE) defined as $\overset{2499}{\underset{i=1}{\sum }}{\left({ŷ}_{i}^{\text{test}}-y_{i}^{\text{PTA}}\right)}^{2}/n$ were computed to evaluate the predictive ability of the two kernels. Here, ${ŷ}_{i}^{\text{test}}$ is the mean of the predictive distribution of response *i* in the testing data set, which is the *i*th element of the $K^{\text{test}↔\text{train}}{\hat{\alpha }}^{\text{train}}$.

## Results

**Figure 2:**
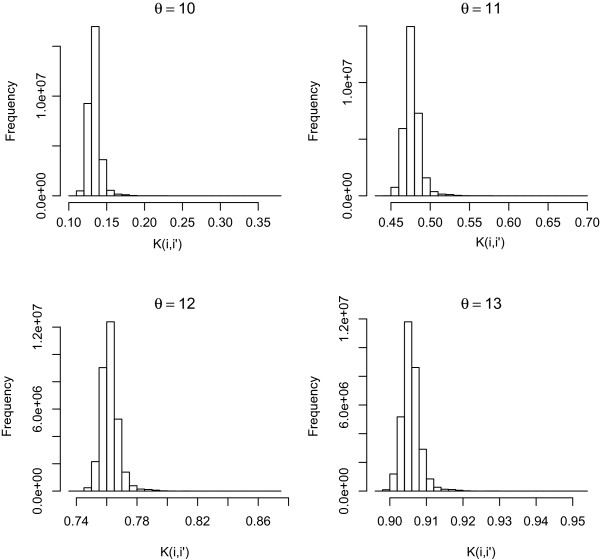
**Histograms of lower triangular elements of four diffusion kernels.** Histograms of lower triangular elements of four diffusion kernels based on four different bandwidth parameters (*θ*).

To illustrate the effect of the bandwidth parameter (*θ*) on the SNP grid kernel, Figure 2 contains histograms showing how *θ* controls similarities among individuals based on evaluating the kernel on the SNP data. The larger *θ* is, the stronger the prior inter-correlation structure. It is important to note that the diagonal elements in our SNP grid kernel matrices are not necessarily equal to one, as opposed to what happens in a Gaussian kernel; here, **K** is a correlation matrix. Table 2 shows the average of diagonal, *K*(*x*_*i*_,*x*_*i*_), and off-diagonal, *K*(*x*_*i*_,*x*_*j*_), elements for diffusion, Gaussian and two additive genomic relationship kernels at varying bandwidth values. The mean values of the diagonal elements of the four diffusion kernels shown in Figure 2 (see Table 2) were 0.369, 0.693, 0.874, and 0.952 for *θ*=10,11,12, and 13, respectively. This is because in equation (13), even when **x**=**x**^′^, so that *n*_1_=*n*_2_=0, *m*_11_ (the number of ‘Aa’ genotypes shared by **x** and **x**^′^) may not be zero. This implies that our diffusion kernel accounts for the degree of heterozygosity in a sample. From the perspective of the kernel as a smoothing function, the diffusion kernel performs smoothing for all elements based on heterozygosity as well as allelic similarity. As explained below, the larger the heterozygosity, the weaker the smoothing, leading to a smaller penalty; this is not the case, however, in the Gaussian kernel. In the kernel computation, each factor in (13) is < 1, and in particular, the factor corresponding to *m*_11_ is the largest. Henceforth, if the sample contains few heterozygotes, our **K** will be large. Consequently, the penalty from the optimizer function *f*, $||f_{ℋ}||=\alpha^{T}K\alpha$, will tend to be large. This is interpretable as imposing stronger smoothing for samples with low heterozygosity. As for the “correlation” with itself, an individual with low heterozygosity will have diagonal elements close to one, as in the case of a Gaussian kernel. Therefore, in addition to the ‘distance’ between genotypes of two individuals, the diffusion kernel takes into account the extent of heterozygosity, while the Gaussian kernel incorporates only the former. Also, the two kernels differ in their definition of distance. The diffusion kernel on the SNP grid is based on the Manhattan distance, while the Gaussian kernel is defined on the Euclidean distance. The Manhattan distance is the distance between two points measured by the the sum of the absolute differences of their coordinates.

**Table 2:** Averages of kernel elements and their predictive correlations for the Holstein data

**Kernel**	***θ***	***k*(*x***_***i***_**,*x***_***i***_**)**	***k*(*x***_***i***_**,*x***_***j***_**)**	**Cor (*ŷ***^***test***^**,*y***^***PTA***^**)**	**MSE**
Diffusion	10	0.369 (0.369)	0.138 (0.134)	0.727 (0.726)	215.93 (216.61)
	11	0.693 (0.693)	0.483 (0.477)	0.745 (0.741)	204.36 (208.68)
	11.5	0.801 (0.801)	0.644 (0.639)	0.739 (0.732)	207.93 (212.97)
	12	0.874 (0.874)	0.765 (0.762)	0.739 (0.728)	210.54 (215.08)
	13	0.952 (0.952)	0.907 (0.906)	0.734 (0.725)	211.50 (217.61)
	14	0.982 (0.982)	0.966 (0.965)	0.729 (0.723)	214.29 (218.70)
Gaussian	5×10^−5^	1 (1)	0.237 (0.225)	0.721 (0.702)	220.675 (233.21)
	2×10^−5^	1 (1)	0.551 (0.542)	0.736 (0.733)	213.41 (213.95)
	1×10^−5^	1 (1)	0.749 (0.742)	0.742 (0.736)	210.14 (211.24)
	5×10^−6^	1 (1)	0.866 (0.861)	0.736 (0.729)	210.24 (214.47)
	3×10^−6^	1 (1)	0.917 (0.914)	0.734 (0.726)	211.51 (216.42)
	1×10^−6^	1 (1)	0.971 (0.971)	0.729 (0.724)	214.37 (217.93)
**G1**^∗^	NA	0.992 (1.009)	-0.000126 (-0.000128)	0.729 (0.722)	214.36 (219.27)
**G2**^∗^	NA	0.894 (0.909)	-0.000113 (-0.00012)	0.730 (0.723)	213.64 (218.31)

Averages of diagonal ***k(x***_***i***_***,x***_***i***_***)*** and off-diagonal ***k(x***_***i***_***,x***_***j***_***)*** kernel elements, predictive correlation, and mean-squared error of prediction (MSE) for the diffusion, Gaussian, and two additive genomic relationship kernels (*G*1^∗^ and *G*2^∗^) with different values of the bandwidth parameter ***θ*** for the Holstein data. Values in parentheses were obtained by combining the SNP grid and the hypercube kernels by applying a same bandwidth parameter. *G*1^∗^ and *G*2^∗^ do not involve bandwidth parameters. The best prediction within the same kernel is underlined.

As shown in Table 2, the average of off-diagonal elements of the diffusion kernel was smaller than that of diagonal elements. This is because the first two terms of (13) will be different from zero (*n*_1_,*n*_2_>0) for a pair of individuals. Diffusion kernel evaluations between an individual and itself were always larger than kernels evaluated between pairs, that is, diagonal elements had the largest values for each row of **K**. In the Gaussian kernel, diagonal elements are always equal to 1 and a smaller *θ* value produces a stronger prior correlation. The first type of additive genomic relationship kernel (**G1**^∗^) had the average diagonal and off-diagonal elements close to 1 and 0, respectively, as expected. Similarly, **G2**^∗^ had an average off-diagonal close to 0 but it had smaller average diagonal elements than those of **G1**^∗^.

The right-most columns of Table 2 give the evaluation of predictive ability of the kernels measured as the correlation between predicted values and observed PTA, and MSE of prediction, for several different bandwidth parameters (**G1**^∗^ and **G2**^∗^ do not involve this parameter). The predictive correlation of the diffusion (SNP grid) kernel was best at *θ* = 11, while with the Gaussian kernel this was achieved at *θ* = 10^−5^. Although the averages of diagonal and off-diagonal elements varied substantially with different bandwidth parameters in the diffusion and Gaussian kernels, the influence of this variability on predictive correlations was small. Importantly, no major difference was observed between the diffusion and the Gaussian kernels in terms of predictive performance. Differences among kernels were very minor, probably due to the fact that the response (PTA) is already a smoothed mean based on a large number of daughters of each bull. There was consistency between the correlation and the MSE, in the sense that the value of *θ* with the highest predictive correlation had the smallest MSE. Predictive performance of **G1**^∗^ was only slightly worse than that of the spatial distance kernels with the best bandwidth parameters.

Values in parentheses in Table 2 were obtained by combining the SNP grid kernel from autosomes and the hypercube kernel from allosomes by applying the same bandwidth parameter. Incorporation of X-chromosome information reduced the average off-diagonal elements slightly and deteriorated predictive performance to some extent. The average diagonal and off-diagonal elements remained the same in **G1**^∗^ and **G2**^∗^, but a minor reduction in their predictive abilities was observed.

In the wheat data, the superiority of the spatial distance-based kernels over the additive genomic relationship kernels was clear. Table 3 indicates that the diffusion and Gaussian kernels had the best predictive correlations (MSE) at 0.586 (0.685) and 0.582 (0.686), respectively, whereas those of **G1** and **G2** were 0.518 (0.709) and 0.521 (0.708). This is likely due to picking up non-additive genetic variation that this wheat data harbors. With binary markers, the diagonal elements of the diffusion kernel are always 1, since in equation (16) the Hamming distance *d*(**x**,**x**^′^) is always zero. As with the Holstein data, no apparent difference was observed between the diffusion and the Gaussian kernels.

**Table 3:** Averages of kernel elements and their predictive correlations for the wheat data

**Kernel**	***θ***	***k*(*x***_***i***_**,*x***_***i***_**)**	***k*(*x***_***i***_**,*x***_***j***_**)**	**Cor(*ŷ***^***test***^**,*y***^***train***^**)**	**MSE**
Diffusion	3	1	0.136	0.586	0.685
	3.25	1	0.289	0.580	0.673
	3.5	1	0.466	0.577	0.681
	4	1	0.752	0.547	0.704
	5	1	0.962	0.522	0.721
Gaussian	0.005	1	0.134	0.582	0.686
	0.003	1	0.290	0.579	0.697
	0.002	1	0.434	0.562	0.697
	0.001	1	0.655	0.558	0.703
	0.0005	1	0.809	0.556	0.673
**G1**	NA	2	-0.003	0.518	0.709
**G2**	NA	2	-0.003	0.521	0.708

Average of diagonal ***k(x***_***i***_***,x***_***i***_***)*** and off-diagonal ***k(x***_***i***_***,x***_***j***_***)*** kernel elements, predictive correlation, and mean-squared error of prediction (MSE) for the diffusion, Gaussian, and two additive genomic relationship kernels at different values of the bandwidth parameter ***θ*** for the wheat data. The predictive correlation and the MSE were obtained from a 10-fold cross-validation. Additive genomic relationship kernels (**G1** and **G2**) do not involve bandwidth parameters. The best prediction within the same kernel is underlined.

## Discussion

Arguably, relationships between phenotypes and genotypes may be non-linear and complex [10,15,31]. For this reason, ignoring non-additive effects such as dominance and epistasis in a model may lead to inferior predictive ability of individual phenotypes.

A spatial distance-based kernel non-parametric regression is capable of mapping genotypes to phenotypes in a way that accurately reflects underlying, albeit unknown, relationships. These kernel methods incorporate non-linearity of a predictor set **x** through a nonlinear transformation of **x**, subsequently allowing analysis of the response in terms of features *ϕ*(**x**) in a linear way. This is particularly useful when the response has a linear relationship with respect to the parameters, but is non-linear on covariates, such as in the case of polynomial regression.

The predictive ability of kernel-based genetic models depends on the choice of a kernel and associated bandwidth parameter(s). If the two data points lie in the real line, $x,x^{{}^{\prime }}\in R$, it seems reasonable to compute their distance in terms of Euclidean distance. However, SNP genotypes, coded as dummy variables, take a discrete form. Therefore, it may be worthwhile to consider a kernel designed to capture the discrete structure of the input variables. The best predictive kernel and its performance may vary depending on the underlying genetic architecture, quantitative trait loci (QTL) numbers and distribution of effects, data set used, and kernel method applied. Here, we investigated the use of ridge regression with a diffusion kernel to assess if this would enhance predictive ability over that of the Gaussian kernel and two additive genomic relationship counterparts. Kondor and Lafferty [25] obtained promising results when the diffusion kernel was compared with several kernels in classification problems with a set of discrete predictors, and this kernel has been used in a microarray-based gene function prediction problem [38]. Ober et al. [18] used the Matérn covariance function, which contains the Gaussian and the exponential kernel as particular cases. Therein, the smoothing parameter controls the actual form of a kernel, and this is directly driven by sample data. Although they obtained a Gaussian form as a choice of the covariance function, the Matérn function is bounded by the Euclidean norm by definition, which may not be suited for discrete genomic data.

A strength of kernels for structured data is their ability to address similarities between two data points $x,x^{\prime }\notin R$[39]. The diffusion kernel defines the distance between two data points on graphs, namely vertices, and projects this information into a more interpretable space. As shown in the context of modeling linkage disequilibrium [40], various graph structures can be used to represent sets of discrete random variables, such as genotypes. Coupled with the representer theorem, the diffusion kernel allows casting underlying graph structures into a regression on the real line under a Hilbert space. The main idea behind this kernel is the matrix exponentiation of the graph Laplacian. The *p*-dimensional grid graph with vertices representing a vector of genotypes was chosen for the graph structure. Each grid conveys information on similarity in terms of the Manhattan distance. Two vertices **x** and $x^{{}^{\prime }}$ are connected if $x_{i}=x_{i}^{{}^{\prime }}$ for all *i*, except at one coordinate. In the Holstein data, with *n*=7902 and *p* = 42 438, it is unlikely that any of two vertices present in our data are connected. However, what grid graphs embrace is how many “steps” separate a vector of genotypes observed in individual *i* from an observed vector of genotypes in individual *j*. The diffusion kernel and its associated graph structure are free of parametric structures. They are constructed without posing genetic architecture assumptions a priori. For illustration purposes, we used 0, 1, and 2 for allele coding, but these should be interpreted as mere strings. Kernel computation still remains the same no matter what allele coding method is adopted here. The parametric component in our study is the construction of the path graph in equation (6), but it is not relevant to gene action modes. This allows us to build a flexible non-parametric model without making strong assumptions a priori. This is appealing, because we seldom know the underlying genetic architectures of complex traits. As shown in past studies including animals [31] and plants [41], a non-parametric method stands out when prediction of phenotypes is the primal focus.

Our motivation for applying the diffusion kernel stemmed from the assumption that a non-Euclidean distance may be able to more clearly represent genomic similarities. We carried out a matrix exponentiation of two graph Laplacians created from two path graphs (one for SNP and one for binary markers) for this purpose. This yields a kernel based on the Manhattan distance accounting for the heterozygosity that two individuals share. The two spatial distance kernels resulted in better predictive performance than the two additive genomic relationship kernels in the wheat data. This agrees with the previous simulation study of Ober et al. [18], in which the Gaussian kernel outperformed **G1** in the presence of non-additive effects. Superiority of the spatial distance kernels was less obvious in the Holstein data. This may be due to the phenotype we chose for this study, since the PTA response variable is a smoothed average using linear mixed models. Although the differences were small in cattle, two non-parametric kernels applied in this study outperformed the additive relationship kernels in two of the datasets used. This suggests that unknown cryptic genetic architectures are likely to be intrinsic to complex traits and, hence, kernels that can accommodate such structure yielded better predictions.

As for the difference between the diffusion and the Gaussian kernels in terms of predictive ability, the diffusion kernel had the highest predictive correlation and the lowest MSE with *θ*=11 in the Holstein data, but the difference with the Gaussian kernel was negligible. The same result was seen in the wheat data. This implies that the Gaussian kernel is robust, even if it incorporates genotypes on the real line such as 1.25 or -12.3. Our objective to properly incorporate genotypes into a kernel had a small impact on predictive ability of yet-to-be observed phenotypes. Although the distance between genotypes is certainly not continuous, additional efforts to discretize the Euclidean distance may not be needed. Another possible reason might be that genotypes do not reside in the Euclidean or non-Euclidean spaces explored here, but in a manifold [27].

Incorporation of X chromosome genotypes for building a kernel led to a smaller average diagonal and off-diagonal elements (to some extent) in spatial distance kernels, but no change was observed in the additive genomic relationship kernels. In both types of spatial distance kernels, however, the predictive correlations were worse than when kernels were constructed purely from autosomes. This suggests that applying specific bandwidth parameters to autosomes and allosomes in the spatial distance kernels might be important. A similar decline in predictive performance was observed in the two additive genomic relationship kernels, which do not involve any bandwidth parameter. Further research is needed to investigate what produces this drop in predictive performance, although if no markers contribute to PL on chromosome X, this would add extra noise.

To our knowledge, this study involves one of the largest data sets employed for spatial kernel-based genome-enabled selection of agricultural species. The challenge here was computation of the diffusion kernel, rather than the Gibbs sampler. Approximately, it took four days to compute one diffusion kernel on a Linux workstation equipped with the Intel(R) Xeon(R) CPU E5450 3.00GHz and 16GB of RAM. The Gaussian kernel required slightly less time for building, but with several candidates over a grid of values of the bandwidth parameter *θ*, this was an expensive task for both kernels. One useful approach might be that of multiple kernel learning (MKL) [36,42], which uses a few kernels with different covariance structure in a single RKHS model. Finally, the SNP grid graph and the hypercube graph used in this study are naive graph structures for modeling discrete inputs. Perhaps developing a graph structure that is more suitable for SNP data might increase predictive correlations.

## Conclusions

We investigated the performance of a diffusion kernel, which was specifically developed to model discrete marker inputs, using Holstein cattle and wheat data. On the one hand, the predictive ability of the diffusion kernel was similar to that of non-spatial distance-based additive genomic relationship kernels in the Holstein data, due to the fact that the response (PTA) is already a smoothed mean based on a large number of daughters of each bull, but outperformed the latter in the wheat data. On the other hand, only minor difference was observed between the diffusion and the Gaussian kernels in terms of predictive performance. Although the diffusion kernel as a choice of basis function may have potential for use in whole-genome prediction, the results of this study suggest that the simple Gaussian kernel is robust enough, and that the scope for enhancing predictive ability via kernel refinement may be limited.

## Appendix A

### Connection between a diffusion kernel and a Gaussian kernel

Intuitively, consider again (4) with a one locus case. In order to make the space continuous, an infinite number of ‘fake’ genotypes between and outside of 0 and 2 are needed. That is, instead of the discrete graph 0−1−2, the interval between 0 and 2, and also outside of it, will be viewed as a ‘continuous’ graph containing genotypes such as 1.23 or −10.5, for example. While the fundamental structure of the graph remains the same, each genotype is connected only to its immediate neighbors, that is, each genotype *x* is connected to only two genotypes, *x*+*dx* and *x*−*dx* for some infinitesimal *dx*. Then, **H** in (5) becomes an infinite-dimensional matrix, and *H*(*x*,*x*^′^) is −2 for *x*^′^=*x* and 1 for *x*+*dx*, *x*−*dx*, because each genotype is connected to its neighboring genotypes on both sides. With the vector of genotypes being now infinite-dimensional, **x**=(−*∞*,⋯,*x*−*dx*,*x*,*x*+*dx*,⋯,*∞*), define a function *f* that returns an “influence” of genotypes, **f**=(*f*(−*∞*),⋯,*f*(*x*−*dx*),*f*(*x*),*f*(*x*+*dx*),⋯,*f*(*∞*)). Approximating *dx* by *h*, it can be seen that:

$$\begin{array}{ll} \frac{1}{h^{2}}[H(x,\cdot )\cdot f]=\frac{f(x+h)-2f(x)+f(x-h)}{h^{2}}\; & \;\\ \;=\frac{\frac{f(x+h)-f(x)}{h}-\frac{f(x)-f(x-h)}{h}}{h}\; & \;\\ \;≅f^{\prime \prime }(x),\; & \; \end{array}$$

where $f^{{}^{\prime \prime }}(x=x_{0})$ is the second derivative of *f* evaluated at *x*_0_. Thus, with space continuity, **H** acts like a second derivative [25]. Using this analogy back in (11), we get:

$$\frac{d}{\mathrm{d\theta}}K_{\theta }(x)=\frac{d^{2}}{dx^{2}}K_{\theta }(x).$$

This equation is called the continuous diffusion equation: the first derivative in “time” is equal to the second derivate in “space”. The solution to this partial differential equation (PDE) with a Dirac delta [43] initial condition of concentration on *x*=0, *k*_0_(*x*)=1_*x*=0_, is given by:

$$G_{\theta }(x)=\frac{1}{\sqrt{4\pi \theta }}\exp\left(-\frac{x^{2}}{4\theta }\right).$$

This is a Gaussian density in a one-dimensional space where $\sigma_{e}^{2}=2\theta$ is the variance of the distribution. With the initial condition *K*_0_(*x*)=*f*(*x*), the solution to this PDE is:

$$K_{\theta }(x)=\int_{R}f(x^{\prime })G_{\theta }(x-x^{\prime })dx^{\prime },$$

where *g*_*θ*_(*x*,*x*^′^)=*G*(*x*−*x*^′^) is called a Gaussian kernel with bandwidth *θ*. Thus, the Gaussian kernel is the ‘space’ continuous analogue of the diffusion kernel as described on the graph. This analogy works exactly the same in higher dimensions.

## Appendix B

### Proof of equation (13)

Consider a graph with one locus, Γ_0_; this graph has form 0−1−2. We compute exp(*θ***H**) where exponentiation is defined as the Taylor expansion (10), differing from componentwise exponentiation. For Γ_0_, **H** is given by:

$$\begin{array}{ll} H=\left[\begin{array}{lll} -1 & 1 & 0\\ 1 & -2 & 1\\ 0 & 1 & -1 \end{array}\right]. & \; \end{array}$$

We make use of the eigendecomposition of matrix **H=PDP**^−1^ and take note of the fact that **H**^*n*^=**PD**^*n*^**P**^−1^. Plugging this **H**^*n*^ in (10), we obtain exp(*θ***H**)=**P** exp(*θ***D**)**P**^−1^. Here exp(*θ***D**) becomes simple componentwise exponentiation because **D** is a diagonal matrix of eigenvalues. For this specific matrix,

$$P=\left[\begin{array}{lll} 1 & 1 & 1\\ -2 & 0 & 1\\ 1 & -1 & 1 \end{array}\right],\;\;\;\;D={\left[\begin{array}{lll} -3 & 0 & 0\\ 0 & -1 & 0\\ 0 & 0 & 0 \end{array}\right]}_{.}$$

Thus, the kernel for a one-dimensional grid graph is:

(18) $$\begin{array}{lll} \;\;\;\;\;\;\;K_{\theta } & =\exp(\theta H)\; & \;\\ & =P\exp(\theta D)P^{-1}\; & \;\\ & =\left[\begin{array}{lll} 1 & 1 & 1\\ -2 & 0 & 1\\ 1 & -1 & 1 \end{array}\right]\;\left[\begin{array}{lll} e^{-3\theta } & 0 & 0\\ 0 & e^{-\theta } & 0\\ 0 & 0 & 1 \end{array}\right]{\;\left[\begin{array}{lll} 1 & 1 & 1\\ -2 & 0 & 1\\ 1 & -1 & 1 \end{array}\right]}^{-1}\; & \;\\ & =\frac{1}{6}\left[\begin{array}{lll} e^{-3\theta }+3e^{-\theta }+2 & -2e^{-3\theta }+2 & e^{-3\theta }-3e^{-\theta }+2\\ -2e^{-3\theta }+2 & 4e^{-3\theta }+2 & -2e^{-3\theta }+2\\ e^{-3\theta }-3e^{-\theta }+2 & -2e^{-3\theta }+2 & e^{-3\theta }+3e^{-\theta }+2 \end{array}\right].\; \end{array}$$

Taking the exponential of eigenvalues always yields a positive real value, so if **H** is symmetric, exp(*θ***H**) is positive definite, suggesting that the diffusion kernel is a valid kernel. Expression (18) is symmetric and in particular,

(19) $$\begin{array}{ll} K_{\theta }(x,x^{{}^{\prime }})=\left\{\begin{array}{ll} -2e^{-3\theta }+2 & \text{if}|x_{i}-x_{i}^{{}^{\prime }}|=1\\ e^{-3\theta }-3e^{-\theta }+2 & \text{if}|x_{i}-x_{i}^{{}^{\prime }}|=2\\ e^{-3\theta }+3e^{-\theta }+2 & \text{if}x_{i}=x_{i}^{{}^{\prime }},x^{{}^{\prime }}\neq 1\\ 4e^{-3\theta }+2 & \text{if}x_{i}=x_{i}^{{}^{\prime }}=1 \end{array}\right) & \; \end{array}$$

Computing every entry of K is computationally unfeasible and unnecessary. We only need to compute entries corresponding to the pair of genotypes appearing in the sample. In particular, if *k*_*i*_(*x*_*i*_,*y*_*i*_) is the contribution of the *i*th locus, then:

$$K_{\theta }(x,x^{\prime })=\overset{p}{\underset{i=1}{\prod }}k_{i}(x_{i},x_{i}^{{}^{\prime }}),$$

where $k_{i}(x_{i},x_{i}^{{}^{\prime }})$ is determined by the relationship between *x*_*i*_ and $x_{i}^{{}^{\prime }}$, and can take only one of the four values specified above. Thus we can write $k_{i}(x_{i},x_{i}^{{}^{\prime }})$ as:

$$\begin{array}{lll} & (e^{-3\theta }-3e^{-\theta }+2)\delta_{|x_{i}-y_{i}|=2}+(-2e^{-3\theta }+2)\delta_{|x_{i}-y_{i}|=1}\; & \;\\ & +(e^{-3\theta }+3e^{-\theta }+2)\delta_{x_{i}=y_{i}\neq 1}+(4e^{-3\theta }+2)\delta_{x_{i}=y_{i}=1}\; & \; \end{array}$$

where *δ* is the indicator function. Therefore,

$$\begin{array}{lll} K_{\theta }^{⊗p}(x,x^{\prime }) & \propto \overset{p}{\underset{i=1}{\prod }}\left\{\right)(e^{-3\theta }-3e^{-\theta }+2)\delta_{|x_{i}-x_{i}^{\prime }|=2}\; & \;\\ & \;\;\;\;\;+(-2e^{-3\theta }+2)\delta_{|x_{i}-x_{i}^{\prime }|=1}\; & \;\\ & \;\;\;\;\;+(e^{-3\theta }+3e^{-\theta }+2)\delta_{x_{i}=x_{i}^{\prime }\neq 1}\; & \;\\ & \;\;\;\;\;+(4e^{-3\theta }+2)\delta_{x_{i}=x_{i}^{\prime }=1}\})\; & \; \end{array}$$

This can be simplified by using the fact that:

$$n_{1}+n_{0}+n_{2}=p,$$

so that:

(20) $$\begin{array}{lll} \;K_{\theta }^{⊗p}(x,x^{\prime }) & ={\left(-2e^{-3\theta }+2\right)}^{n_{1}}{\left(e^{-3\theta }-3e^{-\theta }+2\right)}^{n_{2}}{\left(e^{-3\theta }+3e^{-\theta }+2\right)}^{n_{0}-m_{11}}{\left(4e^{-3\theta }+2\right)}^{m_{11}}\; & \;\\ & ={\left(-2e^{-3\theta }+2\right)}^{n_{1}}{\left(e^{-3\theta }-3e^{-\theta }+2\right)}^{n_{2}}{\left(e^{-3\theta }+3e^{-\theta }+2\right)}^{n_{0}-m_{11}}{\left(4e^{-3\theta }+2\right)}^{m_{11}}\; & \;\\ & \cdot \frac{{\left(e^{-3\theta }+3e^{-\theta }+2\right)}^{p}}{{\left(e^{-3\theta }+3e^{-\theta }+2\right)}^{p}}\; & \;\\ & \propto \frac{{\left(-2e^{-3\theta }+2\right)}^{n_{1}}{\left(e^{-3\theta }-3e^{-\theta }+2\right)}^{n_{2}}{\left(e^{-3\theta }+3e^{-\theta }+2\right)}^{n_{0}-m_{11}}{\left(4e^{-3\theta }+2\right)}^{m_{11}}}{{\left(e^{-3\theta }+3e^{-\theta }+2\right)}^{p}}\; & \;\\ & =\frac{{\left(-2e^{-3\theta }+2\right)}^{n_{1}}{\left(e^{-3\theta }-3e^{-\theta }+2\right)}^{n_{2}}{\left(e^{-3\theta }+3e^{-\theta }+2\right)}^{n_{0}-m_{11}}{\left(4e^{-3\theta }+2\right)}^{m_{11}}}{{\left(e^{-3\theta }+3e^{-\theta }+2\right)}^{n_{0}+n_{1}+n_{2}}}\; & \;\\ & =\frac{{\left(-2e^{-3\theta }+2\right)}^{n_{1}}{\left(e^{-3\theta }-3e^{-\theta }+2\right)}^{n_{2}}{\left(4e^{-3\theta }+2\right)}^{m_{11}}}{{\left(e^{-3\theta }+3e^{-\theta }+2\right)}^{n_{1}+n_{2}+m_{11}}}\; \end{array}$$

Note that one does not need to count *n*_0_.

## Competing interests

The authors declare that they have no competing interests.

## Authors’ contributions

GM conceived, carried out the study, and drafted the manuscript; MK provided mathematical assistance and helped to derive the SNP grid kernel; GJMR and KAW provided critical insights and revised the manuscript; DG supervised the study and revised the manuscript. All authors read and approved the final manuscript.
